# COVID-19 pandemic response fatigue in Africa: causes, consequences, and counter-measures

**DOI:** 10.11604/pamj.supp.2020.37.1.26742

**Published:** 2020-11-16

**Authors:** Olayinka Stephen Ilesanmi, Ayomide Esther Bello, Aanuoluwapo Adeyimika Afolabi

**Affiliations:** 1 Department of Community Medicine, College of Medicine, University of Ibadan, Ibadan, Oyo State, Nigeria,; 2 Department of Community Medicine, University College Hospital, Ibadan, Oyo State, Nigeria,; 3 Department of Pharmacy, University College Hospital, Ibadan, Oyo State, Nigeria

**Keywords:** COVID-19, pandemic fatigue, behavioral change communication, Africa

## Abstract

A notable decline in adherence to COVID-19 preventive measures has been observed despite the increasing number of cases following the suspension of lockdown measures. The African governments have positively responded to the COVID-19 pandemic in previous times, however presently the COVID-19 response on the African continent is in a state of fatigue. Therefore, public vigilance on COVID-19 needs to be reinvigorated through behavioral change communication via different channels of disseminating information. In addition, support systems and social protection should be established to address the COVID-19 pandemic fatigue. Also, increased funding should be made available for enhancing the COVID-19 outbreak response.

## Commentary

The index case of Coronavirus disease (COVID-19) was recorded in Africa on 14^th^ February, 2020 following its declaration as a public health emergency of international concern by the World Health Organization (WHO) on 12^th^ February, 2020 [1]. As of 28^th^ October, 2020, nearly 44 million confirmed cases and 1,168,076 deaths have been recorded globally, with the African continent accounting for 4% each of cases and fatalities [2]. As a part of the COVID-19 response activities, many control measures such as border closure, school closure, restriction of movements, testing and isolation of cases, public health campaigns on social distancing, wearing of face masks, hand hygiene, and nationwide lockdown have been implemented [3]. Following the suspension of lockdown measures, a notable decline in adherence to COVID-19 preventive measures has been observed among many individuals despite the increasing number of cases [4]. The African government has been financially committed towards submerging the COVID-19 pandemic on the continent, However, presently the COVID-19 pandemic response on the African continent seems to wane, a condition termed pandemic fatigue (PF) [3,5]. PF has been defined by the WHO as the lack of motivation to adhere to recommended protective behaviors. PF emerges gradually, and is often impacted by several emotions and perception [6]. Prior to the onset of the COVID-19 pandemic, social distancing, use of face masks, and conscientious handwashing were alien to the general population in Africa. Ordinarily, trying to add few extra steps to our daily routines for a few days can be challenging, however, trying to keep these steps as sustained behavioral change is harder, particularly when no one around us is sick or when the effects of such actions cannot be measured quantitatively. To make up for the difficulty in making sustained behavioral adjustments, humans tend to adopt different coping mechanisms when dire situations drag on for too long and these may result in fatigue and demotivation [7]. The various factors that come into play may include a reduction in the perceived threat from the virus because people are getting used to its existence despite the fact that epidemiological data may suggest an increase in risk [6].

In an issue published on October 6, 2020 by CNBC, the regional director of WHO warned against the increasing level of COVID-19 fatigue in Europe based on aggregated survey data which estimated level of fatigue to be as high as 60% in certain regions [8]. Similarly, anecdotal report from a community survey conducted in Kano State, Nigeria reported that the fear due to COVID-19 has subsided. Many individuals often forget to use their face masks until they come in contact with persons who have symptoms e.g. cough that are suggestive of COVID-19. This mirrors the reaction of the general Nigerian population presently regarding the preventive measures for COVID-19. Apart from certain institutions like hospitals, commercial banks, a visit to places like major markets in cities in Africa presents scenario where wearing a mask seem like a strange thing to do because trading has been on-going just as it was in the pre-pandemic period. Following this train of thought, it is becoming increasingly apparent that the COVID-19 PF poses a major public health risk. Due to the infectious nature of the disease, it is important to note that one's actions impact a greater number of people beyond one's immediate circles. Hence, the effects of every action or inaction are multiplied across board. People are getting more relaxed and less enthusiastic in following the recommended guidelines. Such negligence can result in another surge or spike in the levels of transmission of the diseases which can consequently increase morbidity and mortality rate recorded, as well as increase the risk of re-infection among previously infected people. Collateral damages such as loss of income, lack of access to covid-19 healthcare services were recorded during the pandemic, these can further increase due to new waves of COVID-19, thus resulting in longer recovery time for both individual and national economy.

As the world is gradually relaxing lockdown measures, the surge resulting from fatigue can result in prolonged lockdown measures which directly impacts the socio-economic aspect of the nation. Another important factor that contributes greatly to the PF is the “infodemic” that came with the barrage of information released concerning the pandemic. In Africa, many misconceptions revolved round the COVID-19 pandemic, including the denial of its existence, a political propaganda, a disease of the rich and mighty, and a time-bound illness skewed people's perception and that of those around them. Such misconceptions therefore reduced adherence to the recommended precautions which invariably increased the risk of COVID-19 transmission as well as the continuous spread of false information [9]. Understanding the causes of COVID-19 PF and its potential effects creates a basis for implementing strategies that will forestall such occurrences. According to surveys conducted across different countries of the world, most people have been shown to possess adequate knowledge of COVID-19 and the precautions required to keep safe, yet factors like emotions and context have been found to have greater impact on behaviors than knowledge [9]. This, according to the WHO, implies that strategies involved in providing information and advice alone are not sufficient, rather the strategies should involve policies and interventions, as well as communication. It is also worthy of note that such interventions are not to be a “one-size-fits-all” approach, but dependent on the existing epidemiological situation, behavioral insights, and appropriate societal, cultural and economic considerations [6].

Many high-income countries, including France and Germany have either implemented or preparing for the second phase of the COVID-19 lockdown, African countries should therefore not live in the euphoria that the battle against the COVID-19 outbreak has been concluded. Rather, implemented COVID-19 control measures are to be scaled-up. Firstly, public vigilance on COVID-19 needs to be reinvigorated. Public health campaigns should be intensified to correct the misconception associated with the COVID-19 pandemic. Behavioral change communication must be enhanced through the use of numerous channels of information dissemination such as radio, television, social media, national dailies, magazines etc. In addition, information on the risk factors for COVID-19 transmission should be increasingly available in public places such as markets, motor parks, health facilities, educational institutions, religious worship centers, and organizations. Such risk communication has been described in the Health Belief Model as important for imbibing and sustaining disease-preventive attitude and practices [4]. In addition, overcoming COVID-19 PF highlights the need for further community engagement. The involvement of community stakeholders assures of increased community acceptance of COVID-19 preventive measures such as hand hygiene, use of face masks, and social distancing. Traditional and religious leaders occupy central seats for the enforcement of outlined COVID-19 recommendations, and missing them out on the response activities would certainly be a mishap. When empowered with adequate information on the COVID-19 control measures, community stakeholders could serve as agents of change for intensifying COVID-19 information in their communities. Also, community pharmacists (CP) and patent medicine vendors (PMV) through their critical roles in community-wide health promotion could promote handwashing education, and enhance adherence to the rational use of antimalarial medication among mildly symptomatic COVID-19 patients on home-based care [3,5]. Moreover, CP and PMV could link suspected COVID-19 cases to designated testing centers. All community stakeholders could be empowered to serve as surveillance officers to ensure the accurate reporting of COVID-19 cases and associated fatalities.

Furthermore, flexible and sustainable health plans need to be developed from evidence-based policies for both short and long-term purposes. Such plans would provide a blueprint on the interventions to be embarked upon at each phase of the COVID-19 pandemic in Africa. Such plans would include the creation of virtual support groups, creating opportunities for people to fill their time productively, particularly those that are isolated or have lost their jobs, encouraging habits that relax them and keep them motivated. These plans would also enhance adequate preparedness and help in the asset mapping of experts to function in each of the outlined areas. Adequate mix of capacity in response to identified needs are also required. Increased capacity similarly addresses the need for increased financial commitment towards members of the COVID-19 response team, including expert epidemiologists, IPC team members, and community health volunteers. Moreover, the COVID-19 PF could be prevented by adequate funding to operationalize COVID-19 response plans. Collaborative efforts are required from non-governmental organizations and individuals to support the response activities being championed by the African government. Strategic funds should be commenced by the government early to build strategic shock reserves for the COVID-19 emergency. COVID-19 testing, contact tracing, and public health campaigns are activities which require monetary provision. Also, data-driven knowledge ecosystem should be supported across the African continent. In addition, increasing the testing capacities of regional laboratories should be invested in. Acknowledging and addressing the hardship people experience takes into consideration the fact that the past few months have been trying for all. Surveys have elucidated that the perceived loss related to the lockdown may actually be greater than those directly related to the virus itself [4,10]. Therefore, support systems should also be instituted for providing citizens with palliatives in form of food items and cash to enhance the success of the COVID-19 response activities. In this regard, a reliable supply of medications and technologies are also essential. The development of the COVID-19 vaccine is ongoing; however, a lot of support is needed to ensure its speedy availability.

## Conclusion

As a part of the COVID-19 response activities, many control measures such as border closure, school closure, restriction of movements, testing and isolation of cases, public health campaigns on social distancing, wearing of face masks, hand hygiene, and nationwide lockdown have been implemented. Many high-income countries have embarked on the second COVID-19 lockdown phase, therefore African countries should not rest on their oars amid the COVID-19 outbreak response. Therefore, we recommend that increased funding is made available for enhancing the COVID-19 outbreak response. Also, public vigilance on COVID-19 needs to be reinvigorated through behavioral change communication via different channels of disseminating information. In addition, support systems and social protection through increased provision of monetary and consumable palliatives should be established to address the COVID-19 PF among community members.
